# An unusual case of penetrating head injury in a child

**DOI:** 10.4103/0974-2700.62113

**Published:** 2010

**Authors:** Tanweer Karim, Margaret Topno

**Affiliations:** Department of Surgery, MGM Medical College, Navi Mumbai - 410 209, India

**Keywords:** Penetrating head injury, pediatric neuro-trauma, craniocerebral injury

## Abstract

Penetrating head injuries can be the result of numerous intentional or unintentional events, including missile wounds, stab wounds, and motor vehicle or occupational accidents (nails, screw-drivers). Penetrating head injuries in children constitute only a small part of the total number of traumatic head injuries seen in casualty. We report a case of neuro-trauma who was operated in our institution. Patient, 4 years male presented in casualty on 15/01/09 with a iron rod penetrating into the skull.

## INTRODUCTION

Penetrating head injuries in children constitute only a small part of the total number of traumatic head injuries seen in the casualty. Apart from gunshot and pellet injuries there are a number of household articles that have been described to cause penetrating injuries.

## CASE REPORT

**Figure 1:**
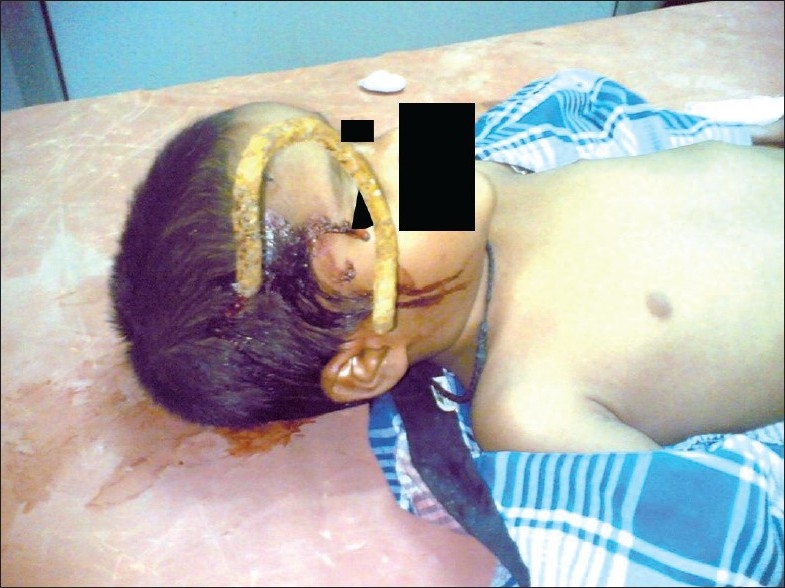
Patient on examination table at the time of initial presentation

We report a case of a 4-year-old male child who presented in the casualty with the history of a fall into a gutter while walking on the road; when he fell, an iron rod penetrated into his head [Figure 1]. There was no history of loss of consciousness or convulsions. At the hospital, the patient was conscious and oriented and responding to verbal commands. His vitals were stable (pulse rate: 100/min, blood pressure: 100/60 mm Hg, and respiratory rate: 18–20/min). The pupils were equal in size and reacting normally to light. The Glasgow coma score (GCS) was 15/15. There was no motor or sensory deficit. Brainstem reflexes were normal. No associated systemic injuries were found.

**Figure 2:**
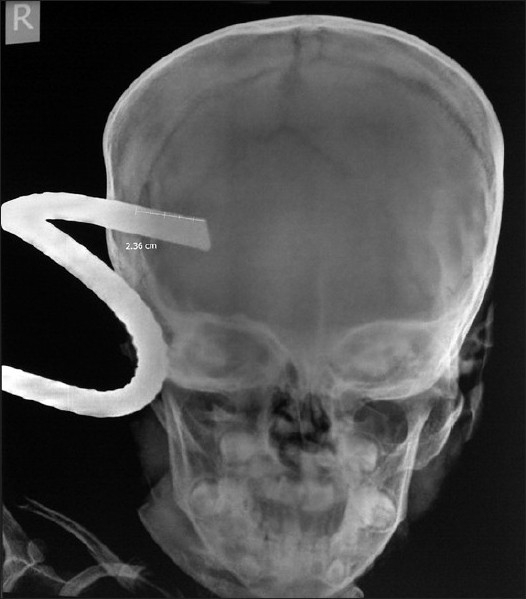
X-ray skull showing iron rod penetrating inside

**Figure 3:**
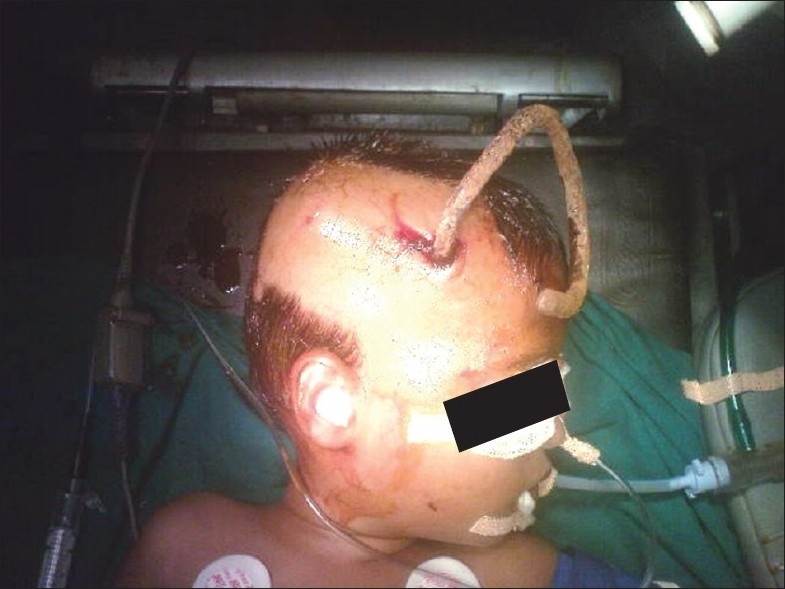
Patient in operation theatre

After primary and secondary evaluation, basic blood investigations and X-ray studies were done of the skull [Figure 2], cervical spine, and chest. Imaging showed that the iron rod (12 mm in diameter) had penetrated approximately 3.5 cm into the brain parenchyma of the right frontoparietal region. A CT scan of the head was not possible immediately because the scanner was out of order. To avoid any delay and aggravation of the injury, the patient was shifted to the operation theater after obtaining written consent [Figure 3]. Circumferential craniectomy was performed and the iron rod was removed along with a bone flap. The tip of the rod was seen to be 3.5 cm inside the brain parenchyma of the right cerebral hemisphere in the region of the precentral gyrus. There was no vascular injury. Necrotic brain tissue, the hematoma, and bone fragments were removed. The wound was closed after debridement of the track. The patient had an uneventful recovery and was able to walk about on day 4. There was no motor or sensory loss and he was able to communicate verbally in a coherent manner.

The absence of major neurological deficits was possible because the patient was right-handed, with left cerebral dominance. CT scan of the head was done on the 6^th^ day to look for intracranial hematoma or abscess formation. The patient had an uneventful recovery and was discharged on the 10^th^ postoperative day with advice to take syp. Gardenal (phenobarbitone), 30 mg at bedtime for 6 months. At the time of discharge his neurological examination was normal. He was advised to come for follow-up monthly and to report immediately if there were any abnormal movements of the body parts or high-grade fever with vomiting and drowsiness. There has been no report of seizure till date. At present he is not receiving any treatment. His growth and development is normal for his age. Police investigation ruled out the possibility of child abuse.

## DISCUSSION

Head trauma is exceedingly common in children, but rarely presents as a penetrating injury of the skull. Most of these injuries are due to a fall.[1] Penetrating head injuries caused by foreign bodies other than bullet and shrapnel are extremely unusual. The most common is due to knife injury, although bizarre cranio-cerebral perforating injuries have been reported, for example those caused by nails, metal poles, ice picks, keys, pencils, chopsticks, and power drills.[2–7] Low-velocity injuries differ from gunshot and missile injuries in that they do not cause concentric zones of cavitations and necrosis. Instead, the damage is predominantly restricted to hemorrhagic infarction in the line of the wound track.[8] They are also very unlikely to have contrecoup injuries and diffuse axonal injury. Thus, in the absence of damage to vital centers and large vessels, the prognosis is usually favorable and this increases the importance of early treatment in these cases to avoid delayed vascular, infectious, and epileptic complications. Early diagnosis is based on clinical evaluation, X-ray skull, and CT scan. MRI can be dangerous in cases of retained ferromagnetic objects due to possible movement in response to the magnetic torque. Nonmissile injuries should undergo a preoperative angiogram to rule out any vascular injury. In penetrating craniocerebral trauma, surgical intervention is aimed at accomplishing the following [9]:

1. Evacuation of masses such as epidural, subdural, or intracerebral hematomas
2. Debridement to remove necrotic tissue, metal, bone fragments, or other foreign bodies to prevent infections
3. Hemostasis
4. Meticulous dural and scalp closure

Complications of penetrating head injuries are infection and seizures. The infection rate is higher in patients with retained bone fragments.[10] Less common complications are cerebrospinal fluid fistula and neuro-endocrine dysfunction.

To conclude, penetrating head injuries in children constitute only a small part of the total number of traumatic head injuries seen in the emergency room. It is a serious injury that may lead to irreversible brain damage and death. A focal neurological deficit may be absent if the non-eloquent area of the brain is involved. There is no doubt that the ‘first golden hour’ following trauma is important in both adults and pediatric patients. Our patient survived the fall and the penetrating injury because he was operated upon promptly.

## References

[1] Atabaki SM (2007). Pediatric head injury. Pediatr Rev.

[2] Pascual JM, Navas M, Carrasco R (2009). Penetrating ballistic- like frontal brain injury caused by a metallic rod. Acta Nurochirurgica.

[3] Salar G, Costella GB, Mottaran R, Mattana M, Gazzola L, Munari M (2004). Multiple craniocerebral injuries from penetrating nails. J Neurosurg.

[4] Jennifer K, Reza K (2001). Penetrating head injury in children: A case report and review of the literature. J Emerg Med.

[5] Bakay L, Glausuer FE, Grand W (1977). Unusual intracranial foreign bodies: Report of five cases. Acta Neurochir (Wien).

[6] Herring CJ, Lumsden AB, Tindall C (1988). Transcranial stab wounds: A report of three cases and suggestions for management. Neurosurgery.

[7] Nakayama Y, Tanaka A, Arita T, Kumate S, Yoshinga S (1995). Penetrating head injury caused by weed: Case report. Brain and Nerve.

[8] Domingo Z, Peter JC, de Villiers JC (1994). Low-velocity penetrating craniocerebral injury in childhood. Pedia Neurosurg.

[9] Trask TW, Narayan RK, Narayan R, Wilberger J, Povlishock J (1996). Civilian Penetrating Head Injury. Neurotrauma.

[10] Hagan RE (1971). Early complications following penetrating wounds of the brain. J Neurosurg.

